# Learning Collaboratives: a Strategy for Quality Improvement and Implementation in Behavioral Health

**DOI:** 10.1007/s11414-022-09826-z

**Published:** 2022-12-20

**Authors:** Heather J. Gotham, Manuel Paris, Michael A. Hoge

**Affiliations:** 1 Mental Health Technology Transfer Center Network Coordinating Office, Stanford University School of Medicine, 1520 Page Mill Road, Palo Alto, CA 94304 USA; 2 The Annapolis Coalition on the Behavioral Health Workforce & Yale University School of Medicine, 34 Park Street, New Haven, CT 06511 USA; 3 The Annapolis Coalition On the Behavioral Health Workforce, & Yale University School of Medicine, 300 George Street, Suite 901, New Haven, CT 06511 USA

## Abstract

Learning collaboratives are increasingly used in behavioral health. They generally involve bringing together teams from different organizations and using experts to educate and coach the teams in quality improvement, implementing evidence-based practices, and measuring the effects. Although learning collaboratives have demonstrated some effectiveness in general health care, the evidence is less clear in behavioral health and more rigorous studies are needed. Learning collaboratives may contain a range of elements, and which elements are included in any one learning collaborative varies widely; the unique contribution of each element has not been established. This commentary seeks to clarify the concept of a learning collaborative, highlight its common elements, review evidence of its effectiveness, identify its application in behavioral health, and highlight recommendations to guide technical assistance purveyors and behavioral health providers as they employ learning collaboratives to improve behavioral health access and quality.

## Introduction

The last several decades have been marked by growing concerns about the quality of health care. A seminal report at the turn of the century by the Institute of Medicine highlighted that as many as 98,000 people were dying annually from medical errors.^1^ This caught public attention and stimulated a national agenda for improving patient safety. What followed were efforts throughout health care, and behavioral health care, to further develop and disseminate evidence-based practices to enhance the quality and effectiveness of care.^2,3^ Developing an evidence-base on safe and effective practices is an essential first step. However, translating science to practice and improving the quality of care delivered in real world settings has proved to be a daunting challenge.^4^

While first developed in general health care, learning collaboratives, also called quality improvement collaboratives, have been used frequently in behavioral health by providers of training, technical assistance, and services as a strategy to promote quality improvement and implementation of a range of evidence-based practices (e.g., cognitive behavioral therapy for psychosis, integrated services for co-occurring mental health and substance use disorders, school mental health, supported employment, trauma informed care).^5–12^ These collaboratives generally involve bringing together teams from different organizations and using experts to educate and coach them in a quality or implementation project and measure the effects.^13^ Sharing of strategies, data, successes, and obstacles among participating teams is central to the approach.^14^

With the proliferation of learning collaboratives has come wide variability in how they are conducted.^15–17^ Technical assistance providers may be unclear about which components of a learning collaborative are most important, how long it should last, and when to offer a learning collaborative rather than an alternative improvement or implementation strategy (e.g., training workshop, community of practice). Behavioral health care providers may struggle with whether to invest significant staff, time, and financial resources in a learning collaborative, whether a collaborative being offered is adequately designed, and what outcomes to expect.

Learning collaboratives have been the subject of many publications and a more modest number of research studies.^6,7,16^ This commentary stems from a project led by the Annapolis Coalition on the Behavioral Health Workforce for the Mental Health Technology Transfer Center Network to improve knowledge about collaboratives within the behavioral health community and to promote, if warranted, an increase in their effective use in the USA. The specific aims were to clarify the concept of a learning collaborative, highlight its common elements, review evidence of its effectiveness, identify its application in behavioral health, and highlight recommendations to guide technical assistance purveyors and behavioral health providers as they employ learning collaboratives to improve behavioral health access and quality.

## Methods

This commentary integrates the findings from a traditional literature review of the published and gray literature and key informant interviews of experts in the field.

### Literature search and review

A literature search of 16 terms related to learning collaboratives was conducted using Ovid MEDLINE, PsycINFO, AMED, PsycARTICLES, PsycEXTRA, Embase, CINAHL, Google Scholar, and Google with filters set for publications in English from 2010 to 2020. The search included the following terms: learning collaborative(s), quality improvement collaborative(s), quality improvement learning collaborative(s), quality collaborative(s), mental health service collaborative care, mental health service quality, behavioral health learning collaborative(s), behavioral health quality improvement collaborative(s), quality improvement collaborative(s) in behavioral health, evidence-based engagement strategy(ies), multidisciplinary quality improvement team(s), community based quality improvement, learning laboratory(ies), breakthrough series, and Network for the Improvement of Addiction Treatment (NIATx). Reference lists in the initial documents identified were reviewed to find additional resources. One member of the study team (MP) conducted the search and reviewed reference lists, which yielded 640 documents. He performed the initial analysis of this literature, identifying 151 relevant documents and then categorized them by focus (mental health, substance use, general health care, or integration of general health and behavioral health). A second member of the team (MH) conducted a detailed analysis of the relevant literature, identifying the major categories of findings and the national surveys, reviews, and individual studies that offered specific findings within those categories. Sources on learning collaboratives in general health care were examined first, providing a context for review of the more limited resources in behavioral health. The other members of the team then vetted the categorization scheme and the distillation of findings into those categories.

### Key informant interviews

Semi-structured interview questions were developed after the review of the literature clarified topics needing further exploration. Fifteen interviews were conducted involving 17 individuals (see Acknowledgements for names) with substantial knowledge related to learning collaboratives in health or behavioral health, including those focused on diverse populations. Experience within this expert group included founding the learning collaborative movement; managing technical assistance and quality improvement networks; funding and shaping federal policy regarding technical assistance; and conducting research on learning collaboratives, quality improvement, and implementation. One member of the study team (MH) conducted, recorded, and analyzed the recordings, using open coding, which was then reviewed by another author (HG).^18^

The first author (MH) integrated information from the literature review and interview findings. Another author (HG) shaped a conceptual framework that further organized the information into a set of distinct findings and recommendations. National leaders in the provision of behavioral health technical assistance, who comprised a Workforce Development Working Group for the Mental Health Technology Transfer Center Network, then reviewed and commented on a draft study report before its finalization.

## Results

### Learning collaboratives: current knowledge

The following points describe and characterize current knowledge about learning collaboratives based on their extensive use in health care and increasing use in behavioral health, as well as evaluation of those efforts. They are organized by the specific aims of clarifying the concept of a learning collaborative, highlighting common elements and structure, reviewing evidence of effectiveness, and identifying applications in behavioral health.

#### Clarifying the concept of a learning collaborative

The traditional learning collaborative model has been reasonably well-defined, broadly disseminated, and widely adopted. Launched in 1995, the Institute for Healthcare Improvement (IHI) Breakthrough Series (BTS) model is considered the origin of the learning collaborative.^13^ Key elements of a BTS collaborative, as identified by IHI in its early publications, included: selecting a specific improvement topic, recruiting expert faculty, enrolling organizations, providing face-to-face learning sessions, Plan-Do-Study-Act (PDSA) cycles of change, offering technical assistance to organization teams, inter-agency sharing and learning, and summation of results and lessons learned.^13,19,20^ The IHI model became extremely popular and was widely disseminated and adopted nationally and internationally.^13,17^

Currently, the term learning collaborative is often used loosely as a label for efforts at quality improvement and implementation that are often not well-defined or described. As learning collaboratives have proliferated, the term has been used for projects that vary widely from the original BTS model, including different elements, lengths, and frequency of meetings, raising questions about the use of the term and expected efficacy of the project.^15,16^ Confusion and variation stem from a number of sources, including a lack of detail in published studies and descriptions of learning collaboratives.^6^ Variation also occurs as technical assistance providers experiment with collaboratives that are shorter, less intensive, and more virtual to reduce overall cost and burden (Lang, Venkatesh, personal communication).^21^ Technical assistance providers depart from the BTS model when the evidence-base for improving quality is not strong, funding is inadequate, the capacity of participating organizations is a concern, or the project goal is limited to provider skills (Lang, Reid, Venkatesh, personal communication). Finally, it is important to note that a BTS learning collaborative is distinct from learning communities and communities of practice, which tend to be more about individual provider development than organizational change, and more about learning than the collection and analysis of data.^25–27^

#### Common elements and functions

Experts have highlighted the elements of learning collaboratives they deem most important, though limited research has yet to consistently demonstrate the relative impact of any specific element.^20,28–30^ A learning collaborative is a bundle of specific elements, such as the composition of the participating teams, number and format of learning sessions (in-person and/or virtual), use of different types of pre-work, and collection of and feedback about data. A systematic review of 20 studies of learning collaboratives in general health care identified 14 process and structural elements (see Table 1).^28^ Although some elements were used more consistently than others (i.e., in-person learning sessions, PDSA cycles, and collection of data for quality improvement were each used by at least 15 of the 20 studies), research has not disentangled the effectiveness of each element.

**Table 1:** Learning collaborative process and structural elements

Learning collaborative element
In person learning sessions
Plan-Do-Study-Act (PDSA) cycles
Site collection of new data for quality improvement (QI)
Multidisciplinary QI teams
QI team calls involving multiple teams
Email or Web support
Leadership involvement or outreach
External support with data synthesis and feedback
Site review of data and use of feedback
Training for non-QI team staff by the QI team
Pre-work: Convening an expert panel
Pre-work: Organizations demonstrate commitment
Training of non-QI team staff by experts
Length of the collaborative

^*^Adapted from Nadeem and colleagues^28^

The length of learning collaboratives varies greatly, and their optimal length and intensity have not been demonstrated. This issue is controversial, particularly as shortening the experience saves costs and reduces burden on participating agencies and their teams. IHI BTS collaboratives currently last between 18 and 24 months, whereas the average for behavioral health collaboratives, based on a systematic review by Nadeem and colleagues, was 14 months (Laderman, personal communication).^6^ Factors to be considered in setting the length include complexity of the intervention, resources available to the sponsor and participating organizations, and goals to be achieved (Everett, Hoover, personal communication). On the one hand, organizational level change takes time and brief learning collaboratives may limit effectiveness and sustainability (Amaya-Jackson, Becker, Laderman, Reid, personal communication). On the other hand, traditional, lengthy collaboratives may outstrip the resources and attention span of many behavioral health agencies (Gustafson, personal communication).

#### Evidence-base for learning collaboratives

In general health care, research on traditional learning collaboratives, albeit incomplete, suggests some effectiveness in improving provider practices and health outcomes.^5,7,15–17^ A systematic review of studies of BTS-style collaboratives from 1995 through 2014 found that most studies reported significant improvement on at least one clinical process or patient outcome measure with a wide range in the magnitude of changes.^7^ More successful collaboratives addressed straightforward aspects of treatment and the large gap between current practice and the evidence-based practice being implemented. However, the authors noted that many studies were not included in the review due to flaws in design, lack of fidelity to a specific collaborative model, and sparse descriptions of collaborative elements. When other reviews have included a broader variety of learning collaboratives and quality improvement processes, they have found more mixed results and suggest that effectiveness varies by contextual factors; for example, learning collaboratives may be more effective in changing provider rather than patient outcomes, and effectiveness increases when a learning collaborative is paired with other quality improvement or implementation strategies.^5,15–17^ The more that learning collaboratives depart from the fidelity of the traditional BTS model, the less assured one can be of their effectiveness.

Organizational and system change is a complex, difficult process. Multiple reviews in health care and the field of dissemination and implementation science have shown that passive implementation strategies (e.g., distributing published or printed recommendations) and even traditional training strategies (e.g., workshops, lectures) are not effective in changing provider behavior and/or implementing evidence-based practices. ^37–39^ Moreover, using a single implementation strategy to foster practice change is less effective, in general, than multi-component strategies.^40–42^ As is highlighted in Table 1, a learning collaborative is a multi-component strategy. Some research and strong consensus among experts suggest that learning collaboratives are likely more effective in improving provider practices and patient outcomes than low intensity interventions such as lectures, workshops, and webinars (Amaya-Jackson, Becker, Gustafson, personal communication).^30,43,44^ A few studies comparing learning collaboratives to less intensive or single strategies have found promising results, and several larger scale, controlled trials are in process.^30,43–46^

#### Learning collaboratives in behavioral health

In behavioral health, learning collaboratives have been used frequently to address a broad range of topics, including large scale, high-quality applications of the traditional BTS style model. Behavioral health learning collaboratives are described in over 60 publications which report on their perceived value in improving quality and implementing evidence-based practices, with several prominent examples. Over 50 collaboratives on Trauma Focused Cognitive Behavioral Therapy have been conducted by the National Center for Child Traumatic Stress.^4^ The National Center for School Mental Health adapted the BTS model to improve school mental health systems across 14 states.^9^ The Child Health and Development Institute used the BTS model in 15 collaboratives to disseminate and sustain various evidence-based mental health treatments.^47^ Others have focused on behavioral health and primary care integration, supported employment, addiction treatment, and emergency department care.^48–52^ Generally, learning collaboratives used for behavioral health topics, compared to health settings, are not markedly different, although those included in Nadeem and colleagues’ systematic review tended to be shorter in length.^6^

Few controlled studies have evaluated the effectiveness of learning collaboratives in behavioral health, though the body of research is growing.^51,53–55^ Nadeem and colleagues’ systematic review found that among the 20 studies that met review criteria, numerous positive trends were reported on provider and patient outcomes, sustainability, and acceptability of collaboratives to providers.^6^ However, the absence of detailed descriptions of studies and adequate comparison data made it impossible to draw firm conclusions about the effects of learning collaboratives in behavioral health. It appears that the amount and quality of research on behavioral health learning collaboratives is increasing, offering the prospect of improved evidence about their effectiveness.

Health equity has received little attention among learning collaboratives in either general health care or behavioral health.^56,57^ Serious inequities in access and quality of services related to race/ethnicity, gender identity, sexual orientation, income, and other social determinants of health are widely recognized in behavioral health care.^58–61^ Several SAMHSA-funded, disparities-focused technical assistance centers offer a range of training and resources on services for diverse populations, including the National Hispanic and Latino and National American Indian and Alaska Native Mental Health Technology Transfer Centers, Behavioral Health Centers of Excellence on African-American, Aging, and LGBTQ populations, and National Network to Eliminate Disparities in Behavioral Health.^62^ It is becoming seen as an imperative that organizational and policy changes be enacted to achieve behavioral health equity.^63,64^

### Recommendations

Drawing on the appraisal of literature and key informant interviews, the following recommendations are offered for organizations that provide or receive technical assistance. The recommendations focus on the underlying rationale for choosing and using a learning collaborative for any given improvement or implementation initiative, the specifics of how a learning collaborative is conducted, including elements and structure, and issues of evaluation. General recommendations that apply to any improvement or implementation initiative appear first, followed by recommendations specific to learning collaboratives. In addition, Table 2 presents selected best practices in planning and conducting collaboratives summarized from the appraisal of the literature and interviews of experts.

**Table 2:** Learning collaborative best practices

Topic area	Specific best practices
Participant selection	• Emphasize diversity, equity, and inclusion • Utilize an application process with competitive selection and specific criteria for inclusion (Tondora, personal communication)^6^ • Assess applicant capacity, readiness, and commitment
Pre-work requirement and activities	• Communicate expectations in advance (Hoover, personal communication) • Become familiar with each agency and its prior experiences (Nadeem, personal communication) • Assess organizational readiness (Lang, personal communication) • Select the most appropriate collaborative team members and team leaders, clarify their roles, and arrange necessary supports for them (Nadeem, personal communication) • Include persons in recovery and family members in teams and the faculty (Davidson, personal communication) • Clarify the goals of agency leaders for collaborative participation and convey that to the agency team (Gustafson, personal communication)
Maximizing interpersonal interaction	• Prioritize peer-to-peer learning (Reid, personal communication) • Encourage participants to share and steal ideas^9^ • Foster motivation, social pressure, and accountability • Use peer interactions to complement top-down consultation (Hoover, personal communication) • Insist on engagement and participation early and do not allow passive participation (Hoover, personal communication)
In person contacts	• View as prime vehicle for building relationships and a sense of community • Use to promote active participation, protecting participants from the distraction of the workplace • Generate enthusiasm about improving care (Laderman, personal communication) • Prioritize in person contacts when: ○ Participants have no prior experience with learning collaboratives (Lang, personal communication) ○ Agencies are mandated to participate (Davidson, personal communication) ○ Participants are from different health sectors (Lang, personal communication) • If feasible, hold in-person meetings at beginning, middle, and end of collaborative
Virtual contacts	• Take advantage of virtual meetings to expand collaborative access to a larger number of agencies and staff per agency (Orobitg, personal communication) • Decrease individual participant burden by reducing time away from the workplace (Gustafson, personal communication) • Use to decrease cost of agency participation, which is critical for safety-net organizations • Use to reduce the environmental impact of travel (Reid, personal communication) • Use virtual small group breakouts to promote interpersonal relationships (Reid, personal communication) • Review newly published reports of all-virtual collaboratives being refined prior to and during the pandemic (Amaya-Jackson, personal communication)
Creating and measuring change	• Assemble evidence-based practices into an explicit Change Package and adapt it to local needs^49^ • Adopt an explicit model of improvement^69^ • Use checklists to promote effective implementation (Gustafson, personal communication) • Require data collection and reporting early and routinely thereafter to foster the habit of working with data (Hoover, personal communication) • Require PDSA cycles and anticipate participant obstacles in using these effectively • Select clear, practical, mixed methods to measure a limited number of process and outcome variables (Dixon, Gustafson, Reid, personal communication) • Monitor learning collaborative completion and dropout rates^7^
Health equity	• Adopt a health equity lens for all aspects of the collaborative (e.g., population of focus, faculty and participant selection) (Reid, personal communication) • Focus collaboratives on behavioral health conditions that uniquely impact diverse communities (Reid, personal communication) • Make the collaborative accessible and feasible for financially strapped safety-net agencies (Venkatesh, personal communication) • Select goals and change strategies in consultation with the individuals affected by health inequities (Gustafson, personal communication) • Establish a goal to improve the connections of individuals to their communities (Skinstad, personal communication) • Enlist faculty from the diverse population of focus and/or educate faculty in advance about that population (Skinstad, personal communication)

#### For any quality improvement or implementation initiative

Adopt an explicit framework for clarifying goals and selecting the quality improvement or implementation strategy to accomplish those goals. The goals of a quality improvement or implementation project vary, and can include changes in attitudes, knowledge, skills, clinical practice, quality, and/or patient outcomes. The providers and recipients of technical assistance should use an explicit planning framework to clarify their goals (Laderman, personal communication). As one example, the Kirkpatrick Model was developed in the 1950s to promote effective training and has been used extensively in health care education.^72^ It identifies four possible goals: Reaction—whether participants find the experience favorable, engaging, and relevant; Learning—whether participants acquire the intended knowledge, skills, attitudes, confidence, and commitment; Behavior—whether participants apply what they learned; and Results—whether targeted outcomes occur as a result of the intervention.^72^ After clarifying goals, decisions can be made about which process improvement or implementation strategy should be used to achieve those goals (i.e., a training workshop may be appropriate for goals of reaction or learning, whereas a learning collaborative may be more appropriate if the goals are behavior or results). Other frameworks include concept mapping and group model building.^73^

Ensure that actual improvements in health care quality and health outcomes are among the goals. Although lectures, workshops, and webinars can increase awareness, facilitate learning, and shift attitudes, more intensive, multi-component interventions, like learning collaboratives, are likely necessary to change provider practice and improve patient outcomes.^30,43,44^ Improving health care and patient health are the ultimate goals of quality improvement and implementation, and should be well represented among the project goals, especially as the evidence regarding whether learning collaboratives affect change at the patient level is mixed.^5,15–17^

Adopt a model for improvement or logic model that explicitly identifies how planned changes will lead to desired outcomes and how those outcomes will be measured. While the selection of goals occurs at a broad conceptual level, detailed planning is needed to map out the process of change. One example is IHI’s Model for Improvement that guides the technical assistance or service provider through this process.^69^ Other models include Lean, Six Sigma, and NIATx (developed specifically for behavioral health).^74–76^ Another example is a logic model, which is a widely recognized method for visually mapping the link between resources applied, activities planned, and desired outcomes and impact. The W.K. Kellogg Foundation offers a Logic Model Development Guide as a resource for non-profit organizations.^77^

Be guided by the evidence on how to promote improvement and implementation, but do not be paralyzed by imperfections in the evidence. Professional perspectives about the effectiveness of learning collaboratives differ, which came to light during expert interviews. Technical assistance providers highlighted the practical and immediate need to choose among the best available strategies to improve quality and accelerate the adoption of evidence-based practices. Researchers emphasized the imperative for more clarity and fidelity to learning collaborative models and more sophisticated evaluations of their effects. In its current, imperfect state, the body of evidence points technical assistance and service providers toward change strategies, such as learning collaboratives, that are more likely to be effective, though still must be tailored to the unique organizational contexts in which they are applied.^30,43,44^

Make health equity the lens through which all work on improvement and implementation is conducted. Adoption of this recommendation is long overdue. In the words of one expert, “Improvement is not occurring if whole groups of people are being left behind.”^25^ Learning collaboratives can be organized and focused on health equity projects and outcomes, as well as include a health equity perspective in all aspects of their development and evaluation.^63–65^ Multiple frameworks and guides exist to assist health and behavioral health agencies to improve health equity.^56,65,66^ Other specific recommendations related to health equity are listed in Table 2.

Measure the impact of initiatives by examining participant satisfaction, practice change, patient outcomes, cost benefit, and sustainability. A thorough evaluation should measure a variety of outcomes to understand what was done and whether it worked. Relevant outcomes in learning collaborative studies include patient and provider variables, acceptability of the collaborative model to providers, sustainability of the changes achieved, impact of specific elements of the collaborative, and estimates of the cost of the collaborative.^6^ In addition, the Reach, Efficacy, Adoption, Implementation, and Maintenance (RE-AIM) framework is frequently used to organize outcomes at both the patient/intervention level (e.g., Reach refers to how many patients receive the practice) and organization/implementation level (e.g., Adoption refers to how many providers/organizations use the practice).^67^

#### For learning collaboratives specifically

Offer or participate in a traditional IHI BTS learning collaborative for improvement or implementation if there are clear evidence-based practices to be adopted, and sufficient resources, provider interest, and capacity to make this approach viable. While traditional learning collaboratives involve significant investments of money, time, and effort, meaningful change generally requires significant investment (Nadeem, personal communication). If the pre-conditions of an evidence base, resources, interest, and capacity are not met, then a learning collaborative, in its traditional format, may not be the best option.

Make sure that the learning collaborative includes key elements identified by IHI and in systematic reviews of research on collaboratives. Individual elements of learning collaboratives have been identified in theoretical and empirical analyses (see Table 1).^15,28,30^ While the unique impact of these elements has yet to be established, some elements are frequently used, and are well-described in related materials and resources.^13,68,69^When planning and considering participation in a learning collaborative, purveyors and providers should carefully consider which elements are included.^30^

Consider offering or participating in variants of a learning collaborative, like learning networks or learning communities, if the evidence-base is less clear or resources and provider interest are less robust. Learning networks, learning communities, and communities of practice are generally less rigorous, intensive, and data-driven, with a more uncertain link to patient outcomes.^25–27,70^ However, they are widely used, particularly to heighten awareness, promote shared learning, and increase professional development among individual practitioners or agencies with a common interest or focus. The impact of such activities on practice change and patient outcomes is less clear.

Pair time-limited collaboratives with participation in ongoing networks to enhance sustainability. Learning collaboratives promote organization and system change to help sustain improved outcomes over time. A key contribution in the field of behavioral health has involved combining time-limited learning collaboratives to catalyze change with ongoing strategies and structures, such as learning communities, peer networks, and technical assistance centers, to sustain change. These typically function to provide continuous training and technical support, centralized data collection, benchmarking, and peer learning among participating organizations. Exceptional examples are found in the areas of child trauma (The National Child Traumatic Stress Network), evidence-based practices in children’s mental health (Child Health and Development Institute), school-based mental health (National Center for School Mental Health), and supported employment (The Individual Placement and Support [IPS] Employment Center).^4,9,47,50^

Build capacity to offer or participate in collaboratives through internal staff development and partnerships with other organizations. Organizations can be strengthened by developing the competencies of their staff to offer or participate in learning collaboratives. Such staff development builds organizational capacity to set goals, develop strategic plans, organize change management teams, create logic models, conduct tests of change, implement evidence-based practices, improve quality, and measure outcomes. Several organizations offer guides to conducting collaboratives, compilations of change strategies, and PDSA resources. For example, IHI developed the Breakthrough Series College, IHI Open School, conferences, Improvement Coach professional development program, and a quality improvement toolkit. Other examples include the TOOLCIT Curriculum for Learning Collaborative Facilitators by the National Child Traumatic Stress Network, which includes an e-learning course, and Planning and Implementing a Successful Learning Collaborative guide by the New York Department of Health AIDS Institute.^68,69^ Educational and training programs (such as the IHI College) are accessible ways for staff to enhance their knowledge and skills, as are the many other guides, resources, and experiential opportunities related to learning collaboratives. Partnering with other organizations to create and offer collaboratives is often an efficient way to build on internal capacities.

Document learning collaboratives and lessons learned from them in detail to maximize the value of these efforts to the field. For the knowledge base about learning collaboratives to grow, especially in behavioral health, it is imperative that those who offer and participate in quality improvement and implementation efforts document the experience and disseminate that information. This includes providing detail about the collaborative elements used, the processes implemented, and the outcomes achieved. Several sets of guidelines for such documentation exist, including “Name it, Define it, Specify it” in the field of implementation science, and the Standards for Quality Improvement Reporting Excellence (SQUIRE 2.0) from the field of health care improvement.^71,72^ Two learning collaborative-specific strategies include the Agency for Healthcare Research and Quality’s learning collaborative taxonomy and the template for describing any learning collaborative across 14 common dimensions developed by Nadeem et al.^6,73,74^

## Implications for Behavioral Health

The behavioral health field has yet to adequately bridge the enormous gaps in access to high-quality, effective treatment for mental health and substance use disorders.^75,76^ This is especially true for diverse populations.^59–61^ Quality improvement and implementation of evidence-based practices are two related approaches being used to close these gaps. Learning collaboratives have emerged as a frequently employed strategy to assist with these efforts.

As learning collaboratives have proliferated, deviations from the traditional model have become commonplace, introducing confusion about the components of collaboratives and their effects. This commentary summarized information from the literature with wisdom drawn from interviews of experts to support technical assistance providers and behavioral health agencies in better understanding what learning collaboratives are and how to use them most effectively.

While some evidence suggests that the traditional model can change provider practice and improve patient outcomes, more research is needed to determine learning collaboratives’ effects in behavioral health care, their essential elements, and optimal length. However, technical assistance providers and behavioral health agencies should not shy away from learning collaboratives because of imperfections in the evolving research. They can draw inspiration and direction from the many high-quality examples of learning collaboratives that have been conducted in behavioral health and the wealth of available educational and training resources. Best practices, as drawn from the literature review and expert interviews, focus on the importance of maximizing interpersonal interaction (in person and virtually), carefully selecting participating organizations and their change teams, conducting pre-work, and assisting teams in implementing interventions and measuring change. Adopting a health equity lens that shapes every aspect of a collaborative is considered essential.

Learning collaboratives are viewed as a heavy lift, involving significant costs, time, and effort.^66^ In an article on engaging behavioral health providers in learning collaboratives, Jensen-Doss et al. suggested that we must “…reconcile the fact that less intensive training methods, such as one-time workshops, are feasible for participants, yet generally ineffective for creating sustained practice change, whereas more intensive, more effective training methods are challenging for many providers to complete.”^77(p.288–289)^

The role of a technical assistance provider is to select an effective improvement and implementation strategy, as well as the agencies and individuals that can participate effectively in that change effort. At the same time, leaving behind other provider agencies because they have less resources and capacity is not justifiable. Such agencies are more likely serving diverse communities that already suffer from serious health inequities. While creative models and numerous resources inform the use of learning collaboratives and other comprehensive strategies to improve practice and health, more intensive advocacy is crucial to ensuring that our communities and the providers that serve them have the resources and supports necessary to be part of these solutions.

## Data Availability

The de-identified interview data that support this commentary are available from the corresponding author upon reasonable request.

## References

[1] Institute of Medicine. *To Err is Human: Building a Safer Health System*. The National Academies Press, 2000. Available at 10.17226/9728. Accessed 29 October, 2022. 25077248

[2] Institute of Medicine. *Crossing the Quality Chasm: A New Health System for the 21*^*st*^* Century.* The National Academies Press, 2001. Available at 10.17226/10027. Accessed 29 October, 2022.

[3] Institute of Medicine. *Improving the Quality of Health Care for Mental and Substance-Use Conditions.* The National Academies Press, 2006. Available at 10.17226/11470. Accessed 29 October, 2022. 20669433

[4] Amaya-Jackson L, Hagele D, Sideris J, et al. Pilot to policy: Statewide dissemination and implementation of evidence-based treatment for traumatized youth. *BMC Health Services Research.* 2018;18:589. Available at 10.1186/s12913-018-3395-0. Accessed 29 June, 2021. 10.1186/s12913-018-3395-0 PMC6064171 30055619

[5] Franco LM, Marquez L. Effectiveness of collaborative improvement: Evidence from 27 applications in 12 less-developed and middle-developed countries. *BMJ Quality & Safety.* 2011;20(8):658–665. Available at 10.1136/bmjqs.2010.044388. Accessed 29 June, 2021. 10.1136/bmjqs.2010.044388 21317182

[6] Nadeem E, Olin SS, Hill LC, et al. A literature review of learning collaboratives in mental health care: Used but untested. *Psychiatric Services.* 2014;65(9):1088–1099. Available at 10.1176/appi.ps.201300229. Accessed 29 June, 2021. 10.1176/appi.ps.201300229 PMC4226273 24882560

[7] Wells S, Tamir O, Gray J, et al. Are quality improvement collaboratives effective? A systematic review. *BMJ Quality & Safety.* 2018;27(3):226–240. Available at 10.1136/bmjqs-2017-006926. Accessed 29 June, 2021. 10.1136/bmjqs-2017-006926 29055899

[8] Bond GR, Drake RE, Becker DR, et al. The IPS learning community: A longitudinal study of sustainment, quality, and outcome. *Psychiatric Services*. 2016;67(8):864–869. Available at 10.1176/appi.ps.201500301. Accessed 29 October, 2022. 10.1176/appi.ps.201500301 27032661

[9] Connors EH, Smith-Millman M, Bohnenkamp JH, et al. Can we move the needle on school mental health quality through systematic quality improvement collaboratives? *School Mental Health*. 2020;12:478–492. Available at 10.1007/s12310-020-09374-x. Accessed 29 June, 2021. 10.1007/s12310-020-09374-x PMC8315192 34322180

[10] Ford JH, II, Osborne EL, Assefa MT, et al. Using NIATx strategies to implement integrated services in routine care: A study protocol. *BMC Health* *Services Research*. 2018;18(1):431. Available at 10.1186/s12913-018-3241-4. Accessed 29 June, 2021. 10.1186/s12913-018-3241-4 PMC5994046 29884164

[11] Kopelovich SL, Hughes M, Monroe-DeVita MB, et al. Statewide implementation of cognitive behavioral therapy for psychosis through a learning collaborative model*. Cognitive and Behavior Practice*. 2019;26(3):439–452. Available at 10.1016/j.cbpra.2018.08.004. Accessed 29 June, 2021.

[12] Margolies PJ, Broadway-Wilson K, Gregory R, et al. Use of learning collaboratives by the center for practice innovations to bring IPS to scale in New York state. *Psychiatric Services*. 2015;66(1):4–6. Available at 10.1176/appi.ps.201400383. Accessed 29 June, 2021. 10.1176/appi.ps.201400383 PMC5846102 25321348

[13] Institute for Healthcare Improvement. *The Breakthrough Series: IHI’s Collaborative Model for Achieving Breakthrough Improvement*, 2003. Available at: http://www.ihi.org/resources/Pages/IHIWhitePapers/TheBreakthroughSeriesIHIsCollaborativeModelforAchievingBreakthroughImprovement.aspx. Accessed 29 June, 2021.

[14] Hulscher MEJL, Schouten LMT, Grol RPTM, et al. Determinants of quality improvement collaboratives: What does the literature show? *BMJ Quality & Safety.* 2013;22(1):19–31. Available at 10.1136/bmjqs-2011-000651. Accessed 29 June, 2021. 10.1136/bmjqs-2011-000651 22879447

[15] Schouten LMT, Hulscher MEJL, van Everdingen JJE, et al. Evidence for the impact of quality improvement collaboratives: Systematic review. *BMJ.* 2008;336(1491). Available at 10.1136/bmj.39570.749884.BE. Accessed 29 June, 2021. 10.1136/bmj.39570.749884.BE PMC2440907 18577559

[16] Zamboni K, Baker U, Tyagi M, et al. How and under what circumstances do quality improvement collaboratives lead to better outcomes? A systematic review. *Implementation Science.* 2020;15(1):27. Available at 10.1186/s13012-020-0978-z. Accessed 23 January, 2022. 10.1186/s13012-020-0978-z PMC7199331 32366269

[17] Garcia-Elorrio E, Rowe SY, Teijeiro ME, et al. The effectiveness of the quality improvement collaborative strategy in low- and middle-income countries: A systematic review and meta-analysis. *PLoS One.* 2019;14(10):e0221919. Available at 10.1371/journal.pone.0221919. Accessed 23 January, 2022. 10.1371/journal.pone.0221919 PMC6776335 31581197

[18] Corbin J, Strauss A (2014). Basics of Qualitative Research: Techniques and Procedures for Developing Grounded Theory.

[19] Kilo CM. A framework for collaborative improvement: Lessons from the Institute for Healthcare Improvement’s Breakthrough Series. *Quality Management in Health Care*. 1998;6(4):1–13. Available at 10.1097/00019514-199806040-00001. Accessed 23 January, 2022. 10.1097/00019514-199806040-00001 10339040

[20] Nembhard IM. Learning and improving in quality improvement collaboratives: Which collaborative features do participants value most? *Health Services Research*. 2009;44(2 Pt 1):359–378. Available at 10.1111/j.1475-6773.2008.00923.x. Accessed 23 January, 2022. 10.1111/j.1475-6773.2008.00923.x PMC2677044 19040423

[21] Boushon B, Provost L, Gagnon J, et al. Using a virtual breakthrough series collaborative to improve access in primary care. *Joint Commission Journal on Quality and Patient Safety*. 2006;32(10):573–584. Available at 10.1016/s1553-7250(06)32075-2. Accessed 23 January, 2022. 10.1016/s1553-7250(06)32075-2 17066995

[22] Wenger E (1998). Communities of Practice: Learning, Meaning, and Identity.

[23] Li LC, Grimshaw JM, Nielsen C, et al. Use of communities of practice in business and health care sectors: A systematic review. *Implementation Science*. 2009;4:27. Available at 10.1186/1748-5908-4-27. Accessed 29 October, 2022. 10.1186/1748-5908-4-27 PMC2694761 19445723

[24] Stoll L, Louis KS (2007). Professional Learning Communities: Divergence, Depth and Dilemmas.

[25] Nadeem E, Olin SS, Hill LC, et al. Understanding the components of quality improvement collaboratives: A systematic literature review. *Milbank Quarterly.* 2013;91(2):354–394. Available at 10.1111/milq.12016. Accessed 29 June, 2021. 10.1111/milq.12016 PMC3696201 23758514

[26] Wilson T, Berwick DM, Cleary P. What do collaborative improvement projects do? Experience from seven countries. *Joint Commission Journal on Quality and Safety*. 2003;29(2):85–93. Available at 10.1016/s1549-3741(03)29011-0. Accessed 29 October, 2022. 10.1016/s1549-3741(03)29011-0 12616923

[27] Gustafson DH, Quanbeck AR, Robinson JM, et al. Which elements of improvement collaboratives are most effective? A cluster-randomized trial. *Addiction.* 2013;108(6):1145–1157. Available at 10.1111/add.12117. Accessed 29 June, 2021. 10.1111/add.12117 PMC3651751 23316787

[28] Grimshaw JM, Thomas RE, MacLennan G, et al. Effectiveness and efficiency of guideline dissemination and implementation strategies. *Health Technology Assessment.* 2004;8(6):iii–72. Available at 10.3310/hta8060. Accessed 29 June, 2021. 10.3310/hta8060 14960256

[29] Davis D, O'Brien MAT, Freemantle N, et al. Impact of formal continuing medical education: Do conferences, workshops, rounds, and other traditional continuing education activities change physician behavior or health care outcomes? *JAMA.* 1999;282(9):867–874. Available at 10.1001/jama.282.9.867. Accessed 29 June, 2021. 10.1001/jama.282.9.867 10478694

[30] Valenstein-Mah H, Greer N, McKenzie L, et al. Effectiveness of training methods for delivery of evidence-based psychotherapies: A systematic review. *Implementation Science.* 2020;15(1):40. Available at 10.1186/s13012-020-00998-w. Accessed 29 June, 2021. 10.1186/s13012-020-00998-w PMC7251851 32460866

[31] Mills KT, Obst KM, Shen W, et al. Comparative effectiveness of implementation strategies for blood pressure control in hypertensive patients: A systematic review and meta-analysis. *Annals of Internal Medicine.* 2018;168(2):110–120. Available at 10.7326/M17-1805. Accessed 29 June, 2021. 10.7326/M17-1805 PMC5788021 29277852

[32] Powell BJ, Proctor EK, Glass JE. A systematic review of strategies for implementing empirically supported mental health interventions. *Research on Social Work Practice.* 2014;24(2):192–212. Available at 10.1177/1049731513505778. Accessed 29 June, 2021. 10.1177/1049731513505778 PMC4002057 24791131

[33] Trogrlić Z, van der Jagt M, Bakker J, et al. A systematic review of implementation strategies for assessment, prevention, and management of ICU delirium and their effect on clinical outcomes. *Critical Care.* 2015;19(1):157. Available at 10.1186/s13054-015-0886-9. Accessed 29 June, 2021. 10.1186/s13054-015-0886-9 PMC4428250 25888230

[34] Speroff T, Ely EW, Greevy R, et al. Quality improvement projects targeting health care-associated infections: Comparing virtual collaborative and toolkit approaches. *Journal of Hospital Medicine.* 2011;6(5):271–278. Available at 10.1002/jhm.873. Accessed 29 June, 2021. 10.1002/jhm.873 21312329

[35] Lee RM, Barrett JL, Daly JG, et al. Assessing the effectiveness of training models for national scale-up of an evidence-based nutrition and physical activity intervention: A group randomized trial. *BMC Public Health*. 2019;19(1):1587. Available at 10.1186/s12889-019-7902-y. Accessed 29 October, 2022. 10.1186/s12889-019-7902-y PMC6883557 31779603

[36] Aschbrenner KA, Pratt SI, Bond GR, et al. A virtual learning collaborative to implement health promotion in routine mental health settings: Protocol for a cluster randomized trial. *Contemporary Clinical Trials.* 2019;84:105816. Available at 10.1016/j.cct.2019.105816. Accessed 29 June, 2021. 10.1016/j.cct.2019.105816 PMC10047804 31344520

[37] Zubkoff L, Lyons KD, Dionne-Odom JN, et al. A cluster randomized controlled trial comparing virtual learning collaborative and technical assistance strategies to implement an early palliative care program for patients with advanced cancer and their caregivers: A study protocol. *Implementation Science.* 2021;16(1):25. Available at 10.1186/s13012-021-01086-3. Accessed 29 June, 2021. 10.1186/s13012-021-01086-3 PMC7951124 33706770

[38] Lang JM, Randall KG, Delaney M, et al. A model for sustaining evidence-based practices in a statewide system. *Families in Society.* 2017;98(1):18–26. Available at 10.1606/1044-3894.2017.5. Accessed 29 June, 2021.

[39] Meredith LS, Mendel P, Pearson M, et al. Implementation and maintenance of quality improvement for treating depression in primary care. *Psychiatric Services.* 2006;57(1):48–55. Available at 10.1176/appi.ps.57.1.48. Accessed 29 June, 2021. 10.1176/appi.ps.57.1.48 16399962

[40] Vannoy SD, Mauer B, Kern J, et al. A learning collaborative of CMHCs and CHCs to support integration of behavioral health and general medical care. *Psychiatric Services.* 2011;62(7):753–758. Available at 10.1176/ps.62.7.pss6207_0753. Accessed 29 June, 2021. 10.1176/ps.62.7.pss6207_0753 21724788

[41] Becker DR, Drake RE, Bond GR, et al. Best practices: A national mental health learning collaborative on supported employment. *Psychiatric Services.* 2011;62(7):704–706. Available at 10.1176/ps.62.7.pss6207_0704. Accessed 29 June, 2021. 10.1176/ps.62.7.pss6207_0704 21724779

[42] Ford JH, II, Stumbo SP, Robinson JM. Assessing long-term sustainment of clinic participation in NIATx200: Results and a new methodological approach. *Journal of Substance Abuse Treatment.* 2018;92:51–63. Available at 10.1016/j.jsat.2018.06.012. Accessed 29 June, 2021. 10.1016/j.jsat.2018.06.012 PMC6336112 30032945

[43] Schall M, Laderman M, Bamel D, et al. *Improving Behavioral Health Care in the Emergency Department and Upstream*. Institute for Healthcare Improvement, 2020. Available at http://www.ihi.org/resources/Pages/IHIWhitePapers/Improving-Behavioral-Health-Care-in-the-Emergency-Department-and-Upstream.aspx. Accessed 24 February, 2022.

[44] Wiltsey Stirman S, Finley EP, Shields N, et al. Improving and sustaining delivery of CPT for PTSD in mental health systems: A cluster randomized trial. *Implementation Science*. 2017;12(1):32. Available at 10.1186/s13012-017-0544-5. Accessed 24 February, 2022. 10.1186/s13012-017-0544-5 PMC5339953 28264720

[45] Jackson CB, Herschell AD, Scudder AT, et al. Making implementation last: The impact of training design on the sustainability of an evidence-based treatment in a randomized controlled trial. *Administration and Policy in Mental Health*. 2021;48(5):757–767. Available at 10.1007/s10488-021-01126-6. Accessed 24 February, 2022. 10.1007/s10488-021-01126-6 33728558

[46] Stanley B, Labouliere CD, Brown GK, et al. Zero suicide implementation-effectiveness trial study protocol in outpatient behavioral health using the A-I-M suicide prevention model. *Contemporary Clinical Trials*. 2021;100:106224. Available at 10.1016/j.cct.2020.106224. Accessed 24 February, 2022. 10.1016/j.cct.2020.106224 PMC7887031 33220488

[47] Wyatt R, Laderman M, Botwinick L, et al. *Achieving Health Equity: A Guide for Health Care Organizations*. Institute for Healthcare Improvement, 2016. Available at https://www.ihi.org/resources/Pages/IHIWhitePapers/Achieving-Health-Equity.aspx. Accessed 24 February, 2022.

[48] Hill JE, Stephani AM, Sapple P, et al. The effectiveness of continuous quality improvement for developing professional practice and improving health care outcomes: A systematic review. *Implementation Science.* 2020;15(1):23. Available at 10.1186/s13012-020-0975-2. Accessed 29 June, 2021. 10.1186/s13012-020-0975-2 PMC7168964 32306984

[49] Institute of Medicine. *Unequal Treatment: Confronting Racial and Ethnic Disparities in Health Care*. The National Academies Press, 2003. Available at 10.17226/12875. Accessed 29 June, 2021. 25032386

[50] Maura J, Weisman de Mamani A. Mental health disparities, treatment engagement, and attrition among racial/ethnic minorities with severe mental illness: A review. *Journal of Clinical Psychology in Medical Settings.* 2017;24(3–4):187–210. Available at 10.1007/s10880-017-9510-2. Accessed 29 June, 2021. 10.1007/s10880-017-9510-2 28900779

[51] Budhwani H, Hearld K, Chavez-Yenter D. Depression in racial and ethnic minorities: The impact of nativity and discrimination. *Racial and Ethnic Health Disparities*. 2015;2(1):34–42. Available at 10.1007/s40615-014-0045-z. Accessed 29 June, 2021. 10.1007/s40615-014-0045-z 26863239

[52] McKnight-Eily LR, Okoro CA, Strine TW, et al. Racial and ethnic disparities in the prevalence of stress and worry, mental health conditions, and increased substance use among adults during the COVID-19 pandemic — United States, April and May 2020. *MMWR Morbidity and Mortality Weekly Report.* 2021;70:162–166. Available at 10.15585/mmwr.mm7005a3. Accessed 29 October, 2022. 10.15585/mmwr.mm7005a3 PMC7861483 33539336

[53] *Practitioner Training*. Substance Abuse and Mental Health Services Administration, 2022. Available at https://www.samhsa.gov/practitioner-training. Accessed 23 January, 2022.

[54] Alves-Bradford JM, Trinh NH, Bath E, et al. Mental health equity in the twenty-first century: Setting the stage. *Psychiatric Clinics of North America.* 2020;43(3):415–428. Available at 10.1016/j.psc.2020.05.001. Accessed 29 June, 2021. 10.1016/j.psc.2020.05.001 32773071

[55] National Academies of Sciences, Engineering, and Medicine. *Achieving Behavioral Health Equity for Children, Families, and Communities: Proceedings of a Workshop*. Washington, DC: National Academies Press, 2019. Available at https://nap.nationalacademies.org/download/25347. Accessed 29 October, 2022. 31063290

[56] Langley GJ, Moen RD, Nolan KM (2009). The Improvement Guide: A Practical Approach to Enhancing Organizational Performance.

[57] Kirkpatrick JD, Kirkpatrick WK (2016). Kirkpatrick’s Four Levels of Training Evaluation.

[58] Powell BJ, Beidas RS, Lewis CC, et al. Methods to improve the selection and tailoring of implementation strategies. *Journal of Behavioral Health Services & Research.* 2017;44(2):177–194. Available at 10.1007/s11414-015-9475-6. Accessed 29 October, 2022. 10.1007/s11414-015-9475-6 PMC4761530 26289563

[59] Black J, Miller D (2008). The Toyota Way to Healthcare Excellence: Increase Efficiency and Improve Quality with Lean.

[60] Black K, Revere L. Six Sigma arises from the ashes of TQM with a twist. *International Journal of Health Care Quality Assurance Incorporating Leadership in Health Services.* 2006;19(2–3):259–266. Available at 10.1108/09526860610661473. Accessed 29 October, 2022. 10.1108/09526860610661473 16875106

[61] McCarty D, Gustafson DH, Wisdom JP, et al. The Network for the Improvement of Addiction Treatment (NIATx): Enhancing access and retention. *Drug and Alcohol Dependence.* 2007;88(2–3):138–145. Available at 10.1016/j.drugalcdep.2006.10.009. Accessed 29 October, 2022. 10.1016/j.drugalcdep.2006.10.009 PMC1896099 17129680

[62] W.K. Kellogg Foundation. *Logic Model Development Guide*. W.K. Kellogg Foundation, 2004. Available at https://www.wkkf.org/resource-directory/resources/2004/01/logic-model-development-guide. Accessed 29 October, 2022.

[63] Dressler R, Janek H, Sager L, et al. Learning collaboratives in medical education: Exploring the impact of collaboratives’ structure and resources and teams’ experience. *American Journal of Medical Quality.* 2020;35(4):297–305. Available at 10.1177/1062860619877941. Accessed 29 October, 2022. 10.1177/1062860619877941 31581785

[64] Raths, D. Tulane creates learning collaborative on health equity. *Healthcare Innovation*. February 15, 2022. Available at https://www.hcinnovationgroup.com/population-health-management/health-equity/news/21256797/tulane-creates-learning-collaborative-on-health-equity. Accessed 29 October, 2022.

[65] *Pursuing Equity Learning and Action Network*. Institute for Healthcare Improvement. Available at http://www.ihi.org/Engage/Initiatives/Pursuing-Equity/Pages/default.aspx. Accessed 13 July, 2022.

[66] U.S. Department of Health and Human Services. *Behavioral Health Implementation Guide for the National Standards for Culturally and Linguistically Appropriate Services in Health and Health Care*. Office of Minority Health, 2021. Available at https://www.minorityhealth.hhs.gov/Assets/PDF/clas%20standards%20doc_v06.28.21.pdf. Accessed 13 July, 2022.

[67] Glasgow RE, Vogt TM, Boles SM. Evaluating the public health impact of health promotion interventions: The RE-AIM framework. *American Journal of Public Health.* 1999;89(9):1322–1327. Available at 10.2105/ajph.89.9.1322. Accessed 13 July, 2022. 10.2105/ajph.89.9.1322 PMC1508772 10474547

[68] National Child Traumatic Stress Network. *TOOLCIT Curriculum for Learning Collaborative Facilitators*. Online course. National Child Traumatic Stress Network, 2013. Available at https://www.nctsn.org/resources/toolcit-curriculum-learning-collaborative-facilitators. Accessed 23 January, 2022.

[69] New York Department of Health AIDS Institute. *Planning and Implementing a Successful Learning Collaborative.* New York Department of Health AIDS Institute, 2008. Available at https://targethiv.org/sites/default/files/file-upload/resources/Plan_Implement-Learning_Collaborative_2008.pdf. Accessed 23 January, 2022.

[70] *Communities of Practice Approach*. Centers for Disease Control and Prevention, 2022. Available at https://www.cdc.gov/phcommunities/resourcekit/intro/cop_approach.html. Accessed 23 January, 2022.

[71] Proctor EK, Powell BJ, McMillen JC. Implementation strategies: Recommendations for specifying and reporting. *Implementation Science.* 2013;8:139. Available at 10.1186/1748-5908-8-139. Accessed 23 January, 2022. 10.1186/1748-5908-8-139 PMC3882890 24289295

[72] Ogrinc G, Davies L, Goodman D, et al. Squire 2.0 (standards for quality improvement reporting excellence): Revised publication guidelines from a detailed consensus process. *BMJ Quality and Safety.* 2016;25(12):986–992. Available at 10.1136/bmjqs-2015-004411. Accessed 23 January, 2022. 10.1136/bmjqs-2015-004411 PMC5256233 26369893

[73] Nix M, McNamara P, Genevro J, et al. Learning collaboratives: Insights and a new taxonomy from AHRQ’s two decades of experience. *Health Affairs (Millwood).* 2018;37(2):205–212. Available at 10.1377/hlthaff.2017.1144. Accessed 23 January, 2022. 10.1377/hlthaff.2017.1144 29401014

[74] Bailie J, Peiris D, Cunningham FC, et al. Utility of the AHRQ learning collaboratives taxonomy for analyzing innovations from an Australian collaborative. *Joint Commission Journal on Quality and Patient Safety.* 2021;47(11):711–722. Available at 10.1016/j.jcjq.2021.08.008. Accessed 23 January, 2022. 10.1016/j.jcjq.2021.08.008 34538583

[75] Substance Abuse and Mental Health Services Administration. *The Way Forward: Federal Action for a System That Works for All People Living with SMI and SED and Their Families and Caregivers. Report to Congress*. Rockville, MD: Interdepartmental Serious Mental Illness Coordinating Committee, 2017. Available at https://www.samhsa.gov/sites/default/files/programs_campaigns/ismicc_2017_report_to_congress.pdf. Accessed 23 January, 2022.

[76] Substance Abuse and Mental Health Services Administration. *Key Substance Use and Mental Health Indicators in the United States: Results from the 2019 National Survey on Drug Use and Health*. HHS Publication No. PEP20–07–01–001, Rockville, MD: Center for Behavioral Health Statistics and Quality, 2020. Available at https://www.samhsa.gov/data/. Accessed 23 January, 2022.

[77] Jensen-Doss A, Smith AM, Walsh LM, et al. Preaching to the choir? Predictors of engagement in a community-based learning collaborative. *Administration and Policy in Mental Health.* 2020;47(2):279–290. Available at 10.1007/s10488-019-00985-4. Accessed 23 January, 2022. 10.1007/s10488-019-00985-4 31617139

