# Correction to: Learning Collaboratives: a Strategy for Quality Improvement and Implementation in Behavioral Health

**DOI:** 10.1007/s11414-023-09830-x

**Published:** 2023-01-24

**Authors:** Heather J. Gotham, Manuel Paris Jr., Michael A. Hoge

**Affiliations:** 1 Mental Health Technology Transfer Center Network Coordinating Office, Stanford University School of Medicine, CA Palo Alto, USA; 2 The Annapolis Coalition on the Behavioral Health Workforce & Yale University School of Medicine, New Haven, CT USA; 3 The Annapolis Coalition On the Behavioral Health Workforce, & Yale University School of Medicine, New Haven, CT USA

**Correction to: J Behav Health Serv Res**

10.1007/s11414-022-09826-z

The first name of a co-author is misspelled. “Manual” should be “Manuel”.

The original article has been corrected.

